# Chromatin remodeling gene AT-rich interactive domain-containing protein 1A suppresses gastric cancer cell proliferation by targeting *PIK3CA* and *PDK1*

**DOI:** 10.18632/oncotarget.10060

**Published:** 2016-06-15

**Authors:** Qian Zhang, Hai-Bo Yan, Jie Wang, Shu-Jian Cui, Xiao-Qing Wang, Ying-Hua Jiang, Li Feng, Peng-Yuan Yang, Feng Liu

**Affiliations:** ^1^ Minhang Hospital and Institutes of Biomedical Sciences, Fudan University, Shanghai 201199, China; ^2^ Department of Systems Biology for Medicine, School of Basic Medical Sciences, Fudan University, Shanghai 200032, China; ^3^ Department of Chemistry, Fudan University, Shanghai 200433, China; ^4^ College of Bioscience and Biotechnology, Key Laboratory of Crop Genetics and Physiology of Jiangsu Province, Yangzhou University, Yangzhou 225009, China

**Keywords:** ARID1A, SWI/SNF complex, gastric cancer, PIK3CA, PDK1

## Abstract

The tumor suppressor gene AT-rich interactive domain-containing protein 1A (*ARID1A*) was frequently mutated in cancers. The modulation mechanism of *ARID1A* for PI3K/AKT signaling in gastric cancer (GC) remains elusive. Here, we found that depletion of endogenous *ARID1A* enhanced the *in vitro* proliferation, colony formation, cellular growth, nutrient uptake and *in vivo* xenograft tumor growth of GC cells. PI3K/AKT activation by *ARID1A*-silencing was profiled using a phospho-protein antibody array. The phosphorylation of PDK1, AKT, GSK3β and 70S6K, and the protein and mRNA expressions of PI3K and PDK1, were upregulated by *ARID1A*-silencing. Chromatin immunoprecipitation and luciferase reporter assay revealed that ARID1A-involved SWI/SNF complex inhibited *PIK3CA* and *PDK1* transcription by direct binding to their promoters. Serial deletion mutation analyses revealed that the ARID1A central region containing the HIC1-binding domain, but not the ARID DNA-binding domain and the C-terminal domain, was essential for the inhibition of GC cell growth, PI3K/AKT pathway phosphorylation and its transcriptional modulation activity of *PIK3CA* and *PDK1*. The proliferation, cellular growth and glucose consumption of *ARID1A*-deficient GC cells were efficiently prohibited by allosteric inhibitors mk2206 and LY294002, which targeting AKT and PI3K, respectively. Both inhibitors also downregulated the phosphorylation of PI3K/AKT pathway in *ARID1A*-deficient GC cells. Such cells were sensitized to the treatment of LY294002, and AT7867, another inhibitor of AKT and p70S6K. The administration of LY294002 alone inhibited the *in vivo* growth of *ARID1A-* deficient GC cells in mouse xenograft model. Our study provides a novel insight into the modulatory function and mechanism of *ARID1A* in PI3K/AKT signaling in GC.

## INTRODUCTION

Gastric cancer (GC) is the fourth and sixth most common cancer in men and women, respectively. The estimated incidence of GC is around 952,000 cases worldwide, along with 723,000 deaths, according to GLOCOCAN statistics in 2012 (http://globocan.iarc.fr/Pages/fact_sheets_population.aspx). Recently, a novel tumor suppressor *AT-rich interactive domain-containing protein 1A* (*ARID1A*) has been found to be frequently mutated in human cancers, including 8–27% of GCs [1–5], 57% of ovarian clear-cell carcinomas (OCCC) [6], 23–42% of endometrioid carcinomas [7, 8], 17% of Burkitt lymphomas [9], 6.0–8.3% of lymphoma [10, 11], 10–16.8% of liver cancers [12–14] and 3–8% of lung cancers [15–17]. *ARID1A* encodes BRG1-associated factor 250 a (BAF250a), a noncatalytic subunit of the SWItch/Sucrose Non-Fermentable (SWI/SNF) chromatin-remodeling complex [18].

These mutations were prevalent for frameshifts or nonsense mutations, which will lead to mRNA decay, protein miss-folding or domain dysfunction. Loss of *ARID1A* expression is frequent in a variety of cancers, especially in gynecologic cancers [19, 20]. ARID1A/BAF250a was absent in 51% of primary GCs and was significantly associated with poor prognosis [5, 21]. We also found that 24% of GC samples analyzed were ARID1A-negative [22]. However, Kim MS *et al* argued that loss of ARID1A expression was not common in GC [23]. Wiegand *et al* found that ARID1A was lost in 20–22.5% of GCs but not significantly associated with any clinical parameters [24]. The intriguing observations emphasize a need for additional analyses.

*ARID1A* deficiency is associated with cancer cell proliferation and metastasis. Reexpression of *ARID1A* in breast cancer cell line T47D suppressed colony formation in soft agar [25]. Silencing of *ARID1A* in GC cell lines enhanced proliferation, while restoring *ARID1A* expression showed reverse effect [5, 21]. ARID1A/BAF250a collaborated with p53 to regulate *CDKN1A* (p21) and *SMAD3* transcription and tumor growth in gynecologic cancers [20]. ARID1A regulated cell cycle-related genes, such as transcription factor *E2F1* [26], *CCNE1* [5] and *c-MYC* [27, 28]. *ARID1A* silencing increased the migration and invasion abilities of liver cancer cells [13]. We found that ARID1A regulated GC cell migration and invasion by modulation of E-cadherin/β-catenin signaling and epithelial-mesenchymal transition (EMT) [22].

*ARID1A* mutation in cancer tended to occur in a synergistic fashion with *PIK3CA* [5, 8, 29–32]. Silencing of *ARID1A* in glioma, ovarian and colon cancer cells upregulated the phosphorylation of AKT and P70S6K [33–35]. Despite the findings, no further analysis has been performed to get insight into the modulatory mechanism of ARID1A of PI3K/AKT signaling. Given that ARID1A is a transcriptional modulator instead of a protein kinase, the direct targets of ARID1A in PI3K/AKT pathway remains elucidative. Furthermore, the modulation role of AIRD1A in GC needs to be further addressed.

In the present study, we analyzed the ARID1A functions in GC cell proliferation, cellular growth and nutrient consumption *in vitro* and *in vivo*. We performed a phosphorylation profiling of PI3K/AKT signaling in GC cells with *ARID1A* depletion and identified the direct transcriptional targets of ARID1A in PI3K/AKT pathway. We also mapped the essential region of ARID1A protein in the transcriptional regulation of its target genes. We analyzed the *in vitro* and *in vivo* drug responses of GC cells with *ARID1A* depletion.

## RESULTS

### *ARID1A* depletion enhances the proliferation and growth of GC cells

**Figure 1:**
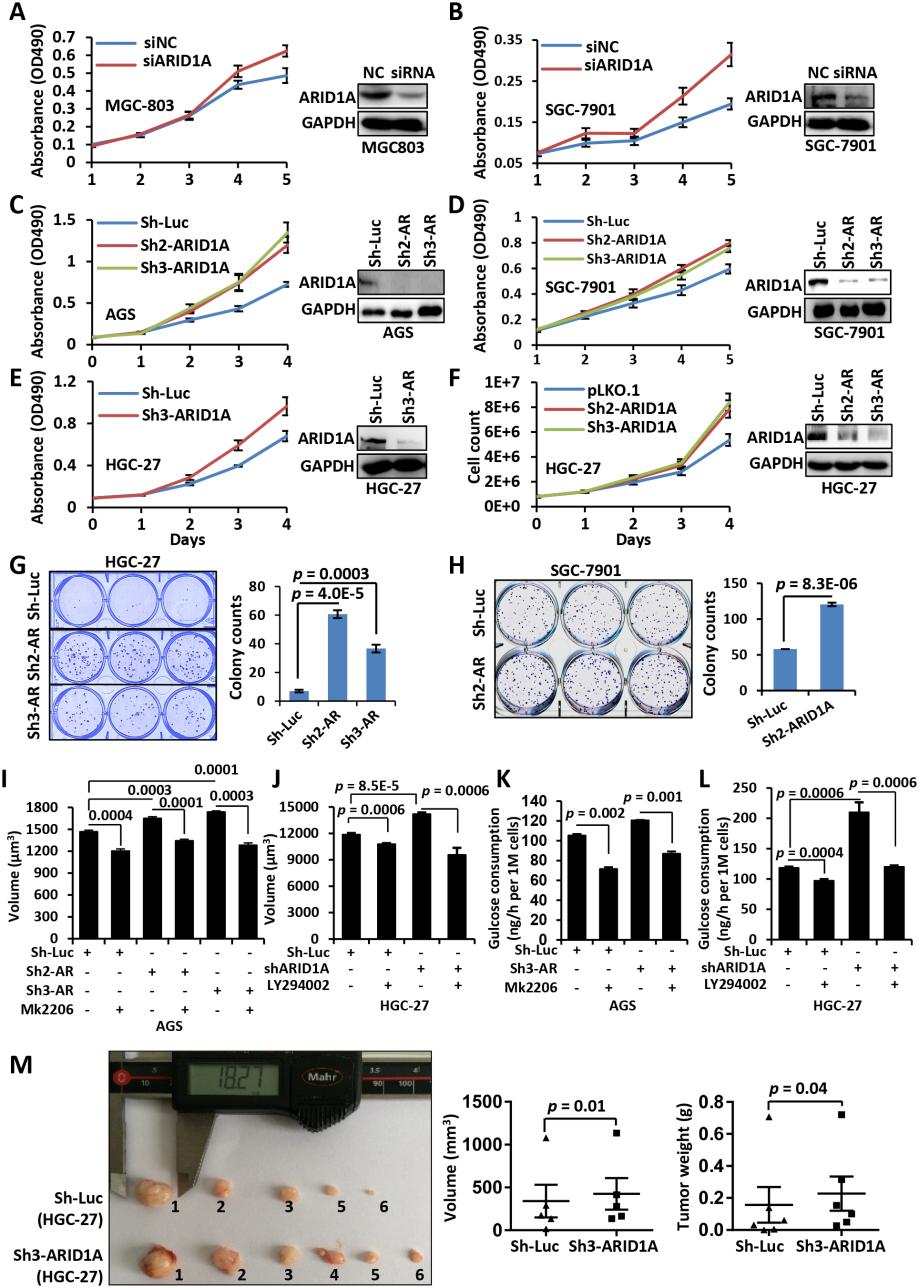
*ARID1A* silencing induces an accelerated proliferation of gastric cancer cells **A, B.** MGC-803 and SGC-7901 cells were transfected with a siRNA targeting *ARID1A* after plating for 24 hrs. After a further culture of 24 hrs, the cells were seeded onto a 96-well plate for growth assay. Cell proliferation was measured using MTT method at day 1, 2, 3, 4 and 5. The Western blot images showed the downregulation of ARID1A in the GC cell lines at day 5. NC, negative control of transfection by scramble siRNA. **C**–**F.** *ARID1A* was stably silenced using shRNAs in GC cell lines AGS, SGC-7901 and HGC-27. AR, ARID1A. Cell proliferation was detected using MTT method (**C**–**E**) or cell count (F). **G, H.** Colony formation assay of GC cell lines HGC-27 and SGC-7901 with *ARID1A* silencing. The colonies were counted manually under a microscopy and the significance was calculated using Student's *t* test. **I.** Cell volume changes of ARID1A-deficient AGS cells under the treatment of mk2206. **J.** Cell volume changes of ARID1A-deficient HGC-27 cells under the treatment of LY294002. **K.** Glucose consumption of AGS cells with ARID1A-silencing under the treatment of mk2206. **L.** Glucose consumption of HGC-27 cells with ARID1A-silencing under the treatment of LY294002. **M.** HGC-27 cells with *ARID1A* silencing were inoculated subcutaneously into both flanks of each nude mice and the transplanted tumors were allowed to develop for 7 weeks. The tumor volume and mice weight were measured every three days. There were six mice used and one control injection (HGC-27-pLKO.1) had no visible tumor developed. The statistics of the final volume and weight of tumors were displayed under the image. The *p* values were calculated using paired, two-sided Student's *t* test.

We silenced endogenous *ARID1A* in GC cell lines MGC-803, AGS, HGC-27 and/or SGC-7901 using a siRNA or shRNAs. The siRNA remained as effective till 5 days post-transfection (Supplementary Figure 1). The proliferation of GC cell lines was enhanced comparing with controls, as revealed by MTT or cell counting method (Figures 1A–1F). The immunofluorescence of Ki-67, a typical nuclear proliferation antigen, was increased in *ARID1A*-depleted cells (Supplementary Figure 2). HGC-27 and SGC-7901 cells with silenced *ARID1A* produced more colonies comparing with controls (Figure 1G and 1H). The average cell sizes (Figure 1I and 1J, Supplementary Figure 3A and 3B) and the glucose consumptions (Figure 1K and 1L, Supplementary Figure 3C and 3D) of GC cells and Hela cells were increased significantly after *ARID1A* knockdown, suggesting that *ARID1A* depletion speeded up nutrients consumption and cellular growth.

To verify the role of *ARID1A in vivo*, we performed a xenograft tumor assay in mice. The HGC-27 cells with *ARID1A* deficiency produced larger (*p* = 0.01) and heavier (*p* = 0.04) tumors than the controls with luciferase silencing (Figure 1M). This *in vivo* assay strengthen the notion that the single gene depletion of *ARID1A* would promote tumor formation.

### *ARID1A* knockdown activates PI3K/AKT phosphorylation cascade in GC cells

**Figure 2:**
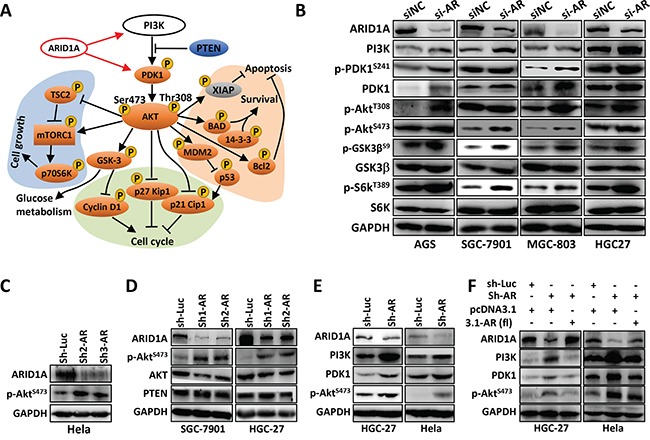
*ARID1A* knockdown activates PI3K-AKT signaling in GC cells **A.** Phospho-protein antibody array analysis of SGC-7901 cells with *ARID1A* silencing. The phospho-proteins of orange were upregulated, while the grey proteins were downregulated. A black arrow indicates activation, whereas a bar at the end of a straight line indicates suppression. The red arrows indicate transcriptional stimulation. **B.** *ARID1A* was transiently silenced in four GC cell lines and the expressions of major components of PI3K-AKT pathway were analyzed. **C.** *ARID1A* was silenced in Hela cell line with shRNAs. AR, *ARID1A*. Luc, luciferase gene. **D**, **E.** *ARID1A* was silenced in SGC-7901, HGC-27 and Hela cells with shRNAs and the expression of ARID1A, BRG1, AKT, p-AKT^S473^, PTEN, PI3K and PDK1 were analyzed. **F.** *ARID1A* was first stably knocked down by a shRNA in HGC-27 and Hela cells, and the expression of ARID1A was further rescued by the ectopic expression of the full-length gene of *ARID1A* (3.1-AR (fl)).

To date, no analysis was performed to profile the phosphorylation changes of PI3K/AKT signaling in GC. The direct target(s) of *ARID1A* in PI3K/AKT pathway remains unclear. Here, we profiled the phosphorylation changes of PI3K/AKT pathway in SGC-7901 cells with *ARID1A* silencing using an AKT/PKB phospho antibody array (Supplementary Figure 4). The phosphorylations of the major components of PI3K/AKT pathway were increased, including AKT, mammalian target of rapamycin (mTOR), glycogen synthase kinase 3α/β(GSK3α/β), p53, p70S6K, PDK1, Bcl-2-associated death promoter (BAD), B-cell lymphoma 2 (BCL-2), tuberous sclerosis complex 2 (TSC2), 14-3-3, p21 and p27 (Figure 2A and Supplementary Table 3). These results indicated that *ARID1A* deficiency in GC cells accelerated cell proliferation and nutrient uptake, but inhibited apoptosis on the other hand. The upregulation of p-AKT^T308^, p-AKT^S473^, p-GSK3^S9^ and p-S6K^T389^ were verified using Western blot (Figure 2B). Interestingly, the total protein expressions of PI3K and PDK1 were upregulated by *ARID1A*-depletion.

Silencing of *ARID1A* increased p-AKT^S473^ in Hela cells (Figures 2C, 2E and 2F) and in HGC-27 and SGC-7901 cells (Figures 2D–2F), while total AKT remained unchanged (Figure 2D). PI3K and PDK1 were upregulated by *ARID1A* depletion (Figure 2E), but were downregulated by *ARID1A* overexpression (Figure 2F). PTEN, a negative regulator of PI3K/AKT pathway, was not changed by *ARID1A* silencing (Figure 2D). Therefore, PI3K and PDK1, but not *PTEN*, might be the targets of ARID1A.

The increased phosphorylation of p21^T145^ and p27^S10/T187^ indicated the dysregulation of cell cycle by *ARID1A* depletion. *ARID1A* silencing increased cell percentage in S phase and broke G2 arrest (Supplementary Figures 5A and 5B). Whereas restored expression of wild-type *ARID1A* in 293FT cell caused G2 arrest and decline of cell percentage in S phase (Supplementary Figure 5C). The expressions of two downstream targets of p53, *CDKN1A* (encoding p21, a negative regulator of cell cycle) and *SMAD3*, were decreased in HGC-27 with *ARID1A* silencing (Supplementary Figures 5D–5F). This observation reinforced previous findings in OCCC that ARID1A might regulate cell cycle in collaboration with p53 [20].

### *PIK3CA* and *PDK1* were the direct transcriptional targets of ARID1A

**Figure 3:**
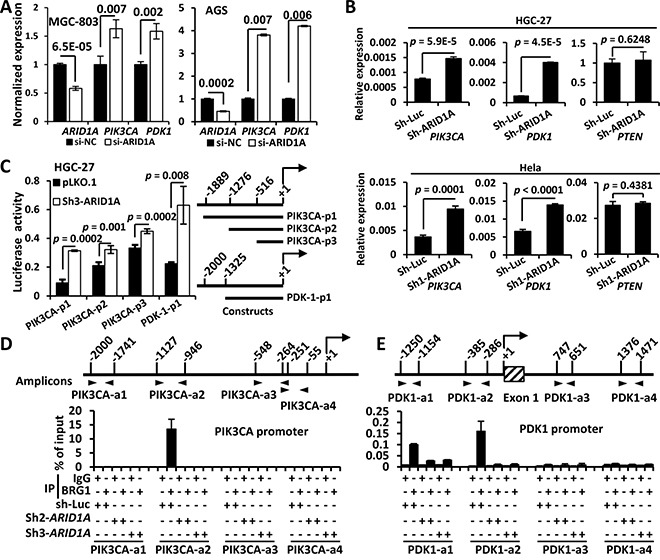
ARID1A negatively regulates *PIK3CA* and *PDK1* transcription by binding to their promoters **A.** The mRNA expressions of *PIK3CA* and *PDK1* were analyzed using quantitative reverse transcription PCR (qPCR) after *ARID1A* was transiently silenced using siRNA. **B.** The mRNA expressions of of *PIK3CA*, *PDK1* and *PTEN* were analyzed using qPCR after *ARID1A* was stably knocked down in HGC-27 and Hela cells using a shRNA. **C.** HGC-27 cells were stably transfected with pLKO.1 (empty vector) and sh3-*ARID1A* and luciferase reporter assays were performed to analyze the activities of different promoter constructs of *PIK3CA* and *PDK1*, which were depicted to the right side. The arrow indicates the transcription start. The numbers indicate the sites (bp) of 5′ terminals of the promoter fragments. **D**, **E.** HGC-27 cells were stably transfected with sh-luciferase (sh-Luc) and shRNAs (*ARID1A*) and ChIP was performed using an antibody against the core catalytic subunit of the SWI/SNF complex, BRG1, or using mouse IgG as a negative control. The PCR amplified regions were illustrated by two opposite arrow heads and the start and end sites were depicted. The box with diagonal lines indicates the first exon of *PDK1* gene.

ARID1A is not a protein kinase, but rather a chromatin-remodeling protein. So we postulate that ARID1A might directly regulate *PIK3CA* and/or *PDK1* at the transcriptional level. As expected, both *PIK3CA* and *PDK1* genes were increased by *ARID1A* silencing with a siRNA or shRNA in GC cells or Hela (Figure 3A and 3B). However, *PTEN* gene remained unchanged, indicating *PTEN* might not be a transcriptional target of ARID1A (Figure 3B).

Dual-luciferase reporter assays of *PIK3CA* and *PDK1* promoters revealed that the luciferase activities of both promoters were increased significantly by *ARID1A* depletion (Figure 3C). We then did a Chromatin immunoprecipitation (ChIP)-PCR assay using an antibody against BRM/SWI2-related gene 1 (BRG1), the core catalytic ATPase subunit of SWI/SNF complex [36]. ARID1A-involved SWI/SNF complex directly interacted with *PIK3CA* promoter within −1127 ∼ −946 bp (Figure 3D). Whereas the binding of the SWI/SNF complex to *PDK1* promoter might occur at regions of −1250 to −1154 and −385 to −286 bp (Figure 3E). These results indicated that *PIK3CA* and *PDK1* were the direct transcriptional targets of ARID1A in PI3K/AKT pathway of GC cells. Thus both *PIK3CA* and *PDK1*, as well as p-AKT, were potential drug targets in *ARID1A*-mutant cancer cells.

### Hypermethylated in cancer 1 (HIC1)-binding domain of ARID1A protein is essential for the modulation of its downstream transcriptional targets

**Figure 4:**
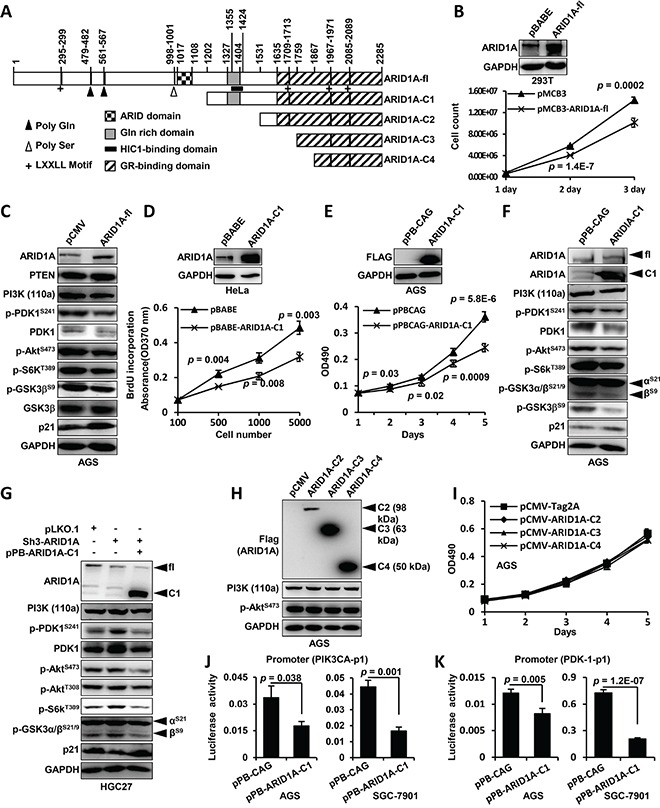
HIC1-interaction domain/Gln domain of ARID1A protein is essential for the modulation of its downstream transcriptional targets **A.** The domain structure of ARID1A protein and the different constructions for ectopic expression were depicted. The positions of domains and motifs were depicted according to the annotation of ARID1A protein (accession O14497, last modified: March 31, 2015) in UniProt database. The amino acid (aa) positions were indicated above the illustration. ARID1A-fl, full-length ARID1A protein. **B.** The full-length ARID1A protein was overexpressed in 293FT cells and cell proliferation was measured by cell counting. **C.** AGS cells were transfected with pCMV or pCMV-ARID1A-fl and the major components of PI3K-AKT signaling pathway were analyzed. The p-GSK3^S9^ was recognized using an antibody against p-GSK3α/β^S21/S9^, and only the band of p-GSK3β^S9^ was shown. **D.** The proliferation of Hela cell transfected with pBABE-ARID1A-C1 was measured using 5-Bromo-2-deoxyuridine (BrdU) incorporation method. **E.** AGS cells were transfected with pPB-CAG-ARID1A-C1 and the proliferation was measured using MTT method. The *p* values at each time point were shown along the curve. **F.** ARID1A-C1 was expressed in AGS cells and the molecules of PI3K-AKT pathway were analyzed. The p-GSK3 was recognized using an antibody against p-GSK3α/β^S21/S9^, and both the p-GSK3α^S21^ and p-GSK3β^S9^ were indicated. **G.** *ARID1A* was first stably silenced in HGC-27 cells and its expression was then rescued by transfecting pPB-CAG-ARID1A-C1 which expressed the C-terminal fragment of ARID1A. fl, full-length protein of ARID1A. C1, C-terminal fragment of ARID1A. **H.** The other three C-terminal fragments of ARID1A were overexpressed in AGS cells and PI3K and p-AKT^S473^ remained unchanged. **I.** The proliferation of AGS cells were measured after the overexpressions of ARID1A-C2, C3 and C4 fragments. **J.** Luciferase reporter assay of the *PIK3CA* promoter (PIK3CA-p1) activity was performed in AGS and SGC-7901 cells transfected with the ARID1A-C1 expression vector. **K.** Luciferase reporter assay was also performed to analyze the *PDK1* promoter (PDK-1-p1) activity in AGS cells and SGC-7901 cells which were transfected with ARID1A-C1 expression vector.

ARID1A protein contains an AT-rich interactive domain (ARID) (aa 1017–1108), a HIC1-binding domain (aa 1355–1424)/Gln domain (aa 1327–1404) [26], a glucocorticoid receptor (GR)-binding domain (aa 1635–2285) [37] and four leucine-rich steroid receptor binding LXXLL motifs (Figure 4A). To map the critical region of ARID1A for transcriptional modulation of *PIK3CA* and *PDK1* in GC cells, we produced deletion mutations of ARID1A protein (Figure 4A). Overexpression of the full-length *ARID1A* (ARID1A-fl) significantly downregulated the proliferation of 293FT cells (Figure 4B) and the levels of PI3K, p-PDK1^S241^/PDK1, p-AKT^S473^, p-S6K^T389^ and p-GSK3β, but increased p21 expression, in AGS cells (Figure 4C). PTEN had no obvious change by *ARID1A*-depletion. ARID1A-C1 overexpression inhibited the proliferations of Hela and AGS (Figure 4D and 4E) and downregulated PI3K/AKT signaling (Figure 4F), suggesting the regulation of PI3K pathway by ARID1A was independent of DNA-binding activity of its ARID domain [38]. *ARID1A* silencing upregulated the activity of PI3K/AKT pathway, while ARID1A-C1 overexpression reduced the activity of the pathway (Figure 4G). Overexpressions of ARID1A-C2, C3 and C4 did not change PI3K and p-AKT^S473^ expressions and GC cell proliferations (Figures 4H–4I). We failed to overexpress the N-terminus of ARID1A (aa 1–1201) in GC cells (data not shown). Luciferase promoter reporter assays revealed that ARID1A-C1 overexpression significantly reduced the activity of promoter construct *PIK3CA*-p1 illustrated in Figure 3C (Figure 4J). Similarly, ARID1A-C1 overexpression downregulated the transcriptional activity of PDK-1-p1 promoter (Figure 3C) in AGS and SGC-7901 cells (Figure 4K). These results suggested that the region from amino acid 1202 to 1531 of ARID1A, which contains the HIC1-binding domain, was essential for the regulation of PI3K/AKT signaling in GC cells.

### MK2206 and LY294002 suppress the proliferation and growth of GC cells with *ARID1A* depletion by deactivation of PI3K/AKT signaling

The above findings suggested that PI3K, PDK1 or p-AKT might be the therapeutic targets of GCs with *ARID1A* deficiency. Given that *PIK3CA* mutation occurs frequently in a synergistic fashion with *ARID1A*, we focused on the drug effectiveness of small inhibitors against *PIK3CA*, as well as p-AKT, which is a “hub” target of diverse tumorigenic signaling. Although the drug sensitivity of *ARID1A*-deficiency has been analyzed in ovarian cancer cell lines [39], no *in vivo* effectiveness of PI3K/AKT inhibitors for GC cells with *ARID1A* depletion has been addressed.

**Figure 5:**
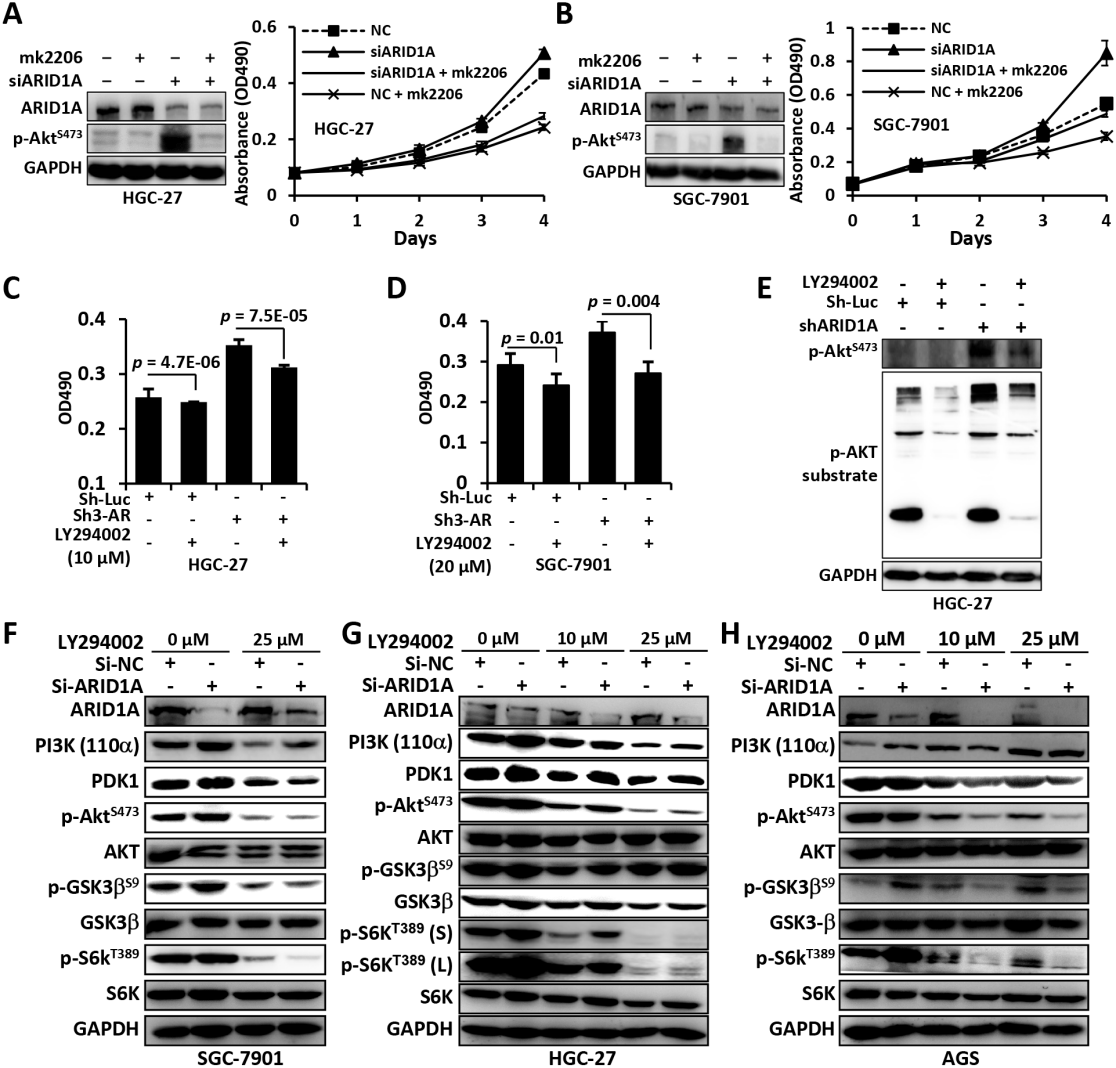
Mk2206 and LY294002 inhibited the proliferation and PI3K/AKT signaling of GC cells with *ARID1A* depletion **A** and **B.** *ARID1A*-depleted HGC-27 and SGC-7901 cells were treated with mk2206, an allosteric inhibitor that inhibits auto-phosphorylation of Akt, and cell proliferation was measured using the MTT method. NC, negative control using a scrambled siRNA. **C** and **D.** *ARID1A* was stably silenced using a shRNA in GC cells and the cells were treated with LY294002, a selective inhibitor of PI3K, and cell proliferation was analyzed using the MTT method. Sh-Luc, shRNA for luciferase. Sh3-AR, Sh3-*ARID1A*. **E.** The substrate activity of p-AKT was analyzed in HGC-27 cells with *ARID1A*-deficiency. **F**–**H.** PI3K-AKT pathway alteration of GC cells with *ARID1A*-depletion under the treatment of different concentration of LY294002. p-GSK3 was detected with an antibody against p-GSK3α/β^S21/9^. p-S6K^T389^ (s) or (l), short exposure or long exposure.

*ARID1A* was silenced in HGC-27 and SGC-7901 cells, which were treated with mk2206, an allosteric inhibitor that inhibits Akt phosphorylation. p-AKT^S473^ and cell growth were obviously upregulated by *ARID1A* depletion but were suppressed by mk2206 treatment (Figure 5A and 5B). The average cell volume and glucose consumption of AGS and Hela cells increased by *ARID1A* silencing, were significantly inhibited by mk2206 (especially for glucose consumption) (Figures 1I and 1K, Supplementary Figure 3B).

We analyzed the inhibitory activity of LY294002, a selective inhibitor of PI3K, for GC cells with *ARID1A* deficiency. The cell proliferation (Figure 5C and 5D), glucose consumption (Figure 1L and Supplementary Figure 3D), cell size (Figure 1J and Supplementary Figure 3B) and p-AKT substrate activity (Figure 5E and Supplementary Figure 3E), were upregulated by *ARID1A* depletion, but were dramatically reduced by LY294002 treatment. The reduction of nutrient uptake by *ARID1A*-deficiency was more prominent than cell volume. Consistently, the upregulation of the major components of PI3K/AKT pathway induced by ARID1A silencing, like PI3K, PDK1, p-AKT^S473^, p-GSK3 and p-S6K^T389^, were inhibited by LY294002 (Figures 5F–5H).

### Sensitivity of GC cells with *ARID1A*-deficiency to drugs targeting PI3K/AKT pathway in mice model

**Figure 6:**
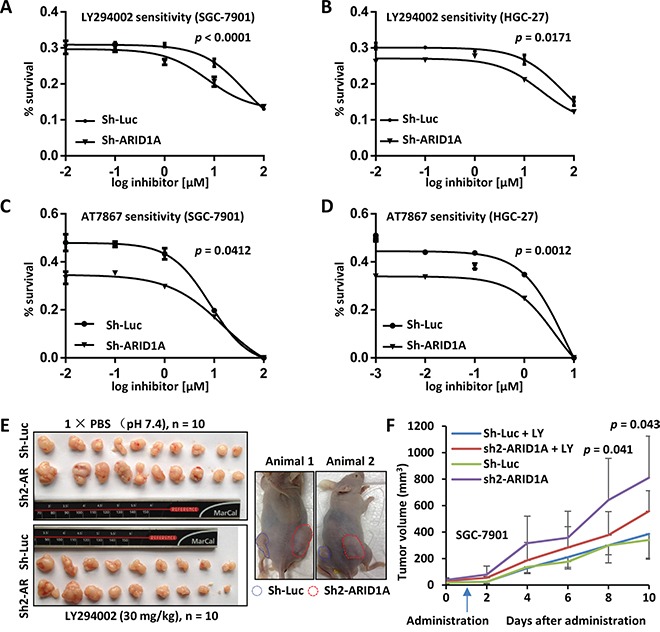
*In vitro* and *in vivo* drug sensitivity analyses of GC cells with *ARID1A*-depletion **A.** SGC-7901 with endogenous *ARID1A*-depletion was sensitized to LY294002 comparing with the control. The *p* value was calculated using F-test and *p* < 0.05 was considered as statistically significant. **B.** Increased sensitivity of HGC-27 with *ARID1A*-silencing to LY294002. **C** and **D.** Increased sensitivity of SGC-7901 and HGC-27 with *ARID1A*-silencing to AT7867, an inhibitor against AKT and p70S6K. **E.** Xenograft mouse model analysis of SGC-7901 with deficient *ARID1A*. The SGC-7901 cells with *ARID1A* knockdown or control gene silencing (Sh-Luciferase) were injected subcutaneously into both flanks of nude mice. After the tumors were established, the mice were treated with LY294002 or PBS as a control by intraperitoneal injection. The mice bearing tumors were shown to the right. Sh-Luc, Sh-Luciferase. Sh-AR, Sh-*ARID1A*. **F.** The xenograft tumors were measured every two days and the volumes were calculated. The arrow indicates the time point the mice received LY294002 treatment. The *p* values above the curve indicate the significant divergence of the volumes between LY294002 and PBS treated tumors of sh2-*ARID1A* cells.

By dose response analysis of LY294002, the LogIC50 was significantly lower in *ARID1A*-depleted SGC-7901 cells (0.8684) than in *ARID1A*-intact cells (1.615) (F test, *p* < 0.0001) (Figure 6A). In HGC-27, *ARID1A*-silenced cells showed a significant low LogIC50 value (1.348) than the control cells with native *ARID1A* (LogIC50 = 1.772) (F test, *p* = 0.0171) (Figure 6B). *ARID1A* silencing also sensitized SGC-7901 (F test, *p* = 0.0412) and HGC-27 (F test, *p* = 0.0012) to AT7867, an inhibitor against AKT and p70S6K (Figure 6C and 6D).

SGC-7901 cells with gene silencing were inoculated into nude mice, which were then treated with LY294002 by intraperitoneally injection after the establishment of tumors (Figure 6E). Interestingly, established tumors with *ARID1A*-depletion were significantly shrunk by LY294002 treatment comparing with mock controls after 8 days of administration (*p* = 0.041 at 8^th^ day and *p* = 0.043 at 9^th^ day) (Figure 6F). However, no obvious changes were observed between the drug treatment and mock treatment of tumors with native *ARID1A*.

These observations suggested that mk2206 and LY294002 targeting PI3K/AKT signaling could effectively suppress the proliferation of GC cells with *ARID1A*-deficiency.

## DISCUSSION

*ARID1A* gene emerges as a bona fide tumor suppressor, while its function and molecular mechanism in GC remains elusive. Although *ARID1A* has been observed to regulate GC cell proliferation by *in vitro* experiments [5, 21], no *in vivo* evidence was established to date. Here we verified the tumorigenesis role of *ARID1A* knockdown in GC by *in vivo* xenograft mouse model and *in vitro* proliferation analyses. Another interesting finding was that *ARID1A* depletion enhanced cellular nutrient uptake and increased average cell size, suggesting *ARID1A* deficiency enhanced tumorous metabolism processes other than accelerating cell cycle, suppressing apoptosis, triggering EMT and increasing cancer cell migration/invasion [20, 22, 27, 40].

In current study, we profiled and verified the phosphorylation of PI3K/AKT pathway in GC cells with *ARID1A* depletion using an antibody array and Western blot. The increased phosphorylations of AKT, PDK1, TSC2, mTORC1 and p70S6K were in line with the enhanced cellular growth of GC cells. The increased phosphorylation of GSK3β was in agreement with the upregulation of glucose consumption, while the phosphorylation of p27 Kip1 and p21 Cip1, two cell cycle inhibitors, were related with accelerated cell cycle. The upregulated phosphorylation of BCL2 were associated with an inhibition of apoptosis [41]. Increased MDM2^S166^ indicated a suppression of p53 function [42]. The increased phosphorylation of BAD^S134/S136^ and 14-3-3 contributed to GC cell survival. These results suggested that the activation of PI3K/AKT pathway by *ARID1A* depletion contributed to multiple cellular alterations including uncontrolled cell cycle, increased survival ability and enhanced nutrient uptake, which had been addressed with additional experiments in current study.

ARID1A is one of the 9 to 12 subunits of SWI/SNF chromatin remodeling complexes which contributed to nucleosome remodeling and transcriptional regulation of a broad profile of target genes [19, 36]. Although the upregulation of p-AKT in cancer cells by *ARID1A* deficiency has been observed, no direct link was established between ARID1A, a transcriptional regulator, and PI3K/AKT pathway, a typical pathway by phosphorylation cascade [31, 33, 39, 43]. In our analysis, we showed evidences that *PIK3CA* and *PDK1* were the direct transcriptional targets of the ARID1A-involved SWI/SNF complex. Furthermore, we found that the negative regulation function of ARID1A for *PIK3CA* and *PDK1* was independent of its N-terminus containing the ARID domain, the LXXLL motifs and its C-terminal fragment containing the GR-binding domain, but interestingly relied on the central region containing the HIC1-interaction domain. ARID domain was known to mediate the binding of ARID1A to DNA sequence in a non-specific fashion [38]. Nie Z *et al* identified that GR-dependent transcriptional activation was reduced slightly by the deletion of the ARID DNA-binding domain but was dramatically reduced by the deletion of its C-terminal GR-binding domain (aa 1637 to 2285) [37]. Here, we showed further evidence that not only the ARID domain but also the GR-binding domain might not have dominant role for its transcription activity, at least in PI3K/AKT signaling in GC cells. Guan B *et al* reported that the C-terminus (aa 1759 to 2285) of ARID1A, the same fragment as ARID1A-C3 in our study, interacted with p53 to regulate the transcription of *p21*, a downstream target gene of p53 [20]. Although the N- or C-terminus of ARID1A was analyzed for its interaction with other proteins, its function in cellular proliferation or growth has not been tested. For the first time, we showed that the C-terminus of ARID1A (1202–2285, ARID1A-C1), but not the shorter forms as C2 (1531–2285), C3 (1759–2285) and C4 (1867–2285), had suppression activity for GC cell proliferation. C3 preserved the activity to bind to p53 as established in 293FT cells by Guan B *et al* [20], however, its inability in GC cell growth inhibition indicated that the binding of ARID1A to p53 might not be essential for its transcriptional activity for target genes other than the genes downstream of p53. HIC1 is a tumor suppressor and a transcriptional repressor [44], which inhibits *E2F1* transcription through the recruitment of SWI/SNF complex by a direct interaction with ARID1A [26]. ARID1A/BAF250a might function as a scaffolding factor through protein-protein interaction that recruiting transcriptional suppressors like HIC1 to promoters of the targeted genes.

As described above, the transcriptional mechanism of ARID1A would be addressed to deal with the heterogeneity of different cancers and physiological and pathological variations. Although ovarian cancer cell lines with *ARID1A*-deficiency showed some *in vitro* sensitivities to PI3K/AKT inhibitors [39], no *in vivo* responsiveness of GC cells with *ARID1A*-deficiency effectiveness of PI3K/AKT inhibitors has been addressed. We analyzed the effects of chemical inhibitors against PI3K/AKT signaling *in vitro* and *in vivo*. Mk2206 and LY294002, which respectively targeting p-AKT^S473^ and PI3K, were effective to downregulate GC cell proliferation, glucose consumption and cellular growth (cell size) conferred by *ARID1A* depletion. Accordingly, the PI3K/AKT signaling was downregulated by mk2206 or LY294002. The drug sensitivity of ARID1A-deficient GC cells was increased for the treatment of LY294002 and AT7867, another inhibitor against AKT and p70S6K. The administration of LY294002 alone inhibited the *in vivo* growth of the xenograft tumors with *ARID1A* silencing, comparing with the cells with native *ARID1A*.

Taken together, our study revealed that deficient *ARID1A* confers an increased proliferation, cellular growth and nutrient consumption of GC cells, companied with an activation of PI3K/AKT signaling. *PIK3CA* and *PDK1* were the direct transcriptional targets of the ARID1A-involved SWI/SNF complex. The central region containing the HIC1-binding domain of ARID1A was essential for its transcriptional regulation of downstream targets. *ARID1A* deficiency and the activation of PI3K/AKT were hazard factors of GC patients and associated with poor prognosis. MK2206 and LY294002 were effective to suppress the proliferation of GC cells with *ARID1A* deficiency by downregulating activated PI3K/AKT signaling. Our study provides a novel insight into the function and mechanism of *ARID1A* in the modulation of PI3K/AKT signaling in GC.

## MATERIALS AND METHODS

### Cell lines

GC cell lines HGC-27 and MGC-803 were purchased from the Cell Bank of Shanghai Institutes for Biological Sciences, China. SGC-7901 was generously provided by Dr. Jian-Jun Du at Huashan Hospital, Fudan University. AGS was a gift by Dr. Qing-Hua Zhang at the Shanghai University of Traditional Chinese Medicine. GC cells were cultured in RPM1640 media supplemented with 10% FBS in a humidified incubator at 37°C and 5% CO_2_. Hela and HEK293FT were cultured in DMEM supplemented with 10% FBS. The cell lines were authenticated on August, 2014.

### Gene cloning and lentivirus production

The *ARID1A* gene was cloned as described previously [22]. The shRNA lentiviral plasmid (pLKO.1) was purchased from the RNAi consortium. The shRNAs of ARID1A had been described previously [22]. Lentivirus was generated in HEK293FT cells using the second-generation packaging system pMD2.G (Addgene plasmid 12259) and psPAX2 (Addgene plasmid 12260). Lentiviral titer was determined as described [45]. The primers and shRNAs used were listed in Supplementary Table 1.

### Western blot analysis

Immunoblotting analysis was performed as previously described [22]. All antibodies used in current study were listed in Supplementary Table 2.

### Immunofluorescence

Immunofluorescence was performed to monitor the proliferation of GC cells after *ARID1A* silencing according to the procedure established previously [46].

### Phospho-protein antibody array

The experiment was performed by Wayen Biotechnologies (Shanghai), Inc., using the Full Moon AKT/PKB phospho antibody array containing 216 antibodies against the corresponding proteins of AKT/PKB pathway (Full Moon BioSystems, Inc.). There are 93 paired antibodies, which react with a phosphorylated site and a paired non-phosphorylated site. Proteins were extracted from SGC-7901 or HGC-27 cells stably transfected with sh-Luciferase or sh2 (*ARID1A*) using the Extraction Buffer (Full Moon BioSystems, Inc.). Next, 25 μg proteins of each sample were labeled with 30 μg biotin in 4% N,N-Dimethylformamide solution. The reaction was stopped after 2 hours of incubation at room temperature. The labeled proteins were diluted with 6 mL Coupling Solution (Full Moon BioSystems, Inc.). The antibody arrays were blocked with the Blocking Reagent (Full Moon BioSystems, Inc.) at room temperature for 45 min and then rinsed 10 times with Milli-Q water. The diluted labeling products were incubated with the antibody arrays at 4°C overnight. The array slides were washed with Milli-Q water as above. The conjugated proteins on the array slides were detected with Cy3-streptavidin (0.5 μg/mL) solution. The slides were scanned using a microarray scanner GenePix 4000B with software GenePix Pro 6.0 (Axon Instruments, USA). The raw data was treated with the Grubbs' algorism and outliers in each six-repeat data point of an antibody were excluded. The data was average for each antibody and all data were normalized using the average against beta-actin. The phosphorylation ratio = phospho/unphospho. The phosphorylation fold change = (phosphorylation sh2-*ARID1A*)/(phosphorylation sh-Luciferase). The proteins with a fold change ≥ 1.8 were reported in Supplementary Table 3. The differential significance were evaluated with two-sided Student's *t* test and a p value < 0.05 was considered as statistically significant.

### Cell cycle assay

Cell cycle alteration by *ARID1A* silencing was measured using the Cell Cycle Detection Kit (Biyuntian Inc. Haimen, China) with a Beckman Flow Cytometer (Beckman Coulter, Inc.). Cells in suspension were fixed by adding 2.5 mL 70% alcohol and incubated at 4°C overnight. Alcohol was aspirated with centrifugation and the cells were washed with PBS/1% BSA triple times and suspended with 50 μg/mL propidium iodide and 0.1 mg/mL RNase. After incubation at 37°C for 40 min, the cells were washed by adding 3 mL PBS/1% BSA. Finally, the cells were re-suspended with 1 mL PBS and analyzed by a cytometer.

### RNA extraction and qPCR

Total RNA isolation and qPCR were performed as previously described [22]. Primers are listed in Supplementary Table 1.

### Colony formation

Colony formation assay was performed as describe previously [47].

### Cell transfection

Plasmid or siRNA transfection using lipofectamine (Life Technologies - Invitrogen) and OPTI-MEM reduced serum media (Life Technologies - Invitrogen) was performed as described previously [46]. The cells were transfected after plating for 24 h. After a further incubation of 24 h, the cells were seeded onto a 96-well plate for growth curve analysis. The sequences of *ARID1A* siRNAs were synthesized as previously reported [22]. The siRNAs were listed in Supplementary Table 1.

### 5-Bromo-2-deoxyuridine (BrdU) assay

Cell proliferation was analyzed using a BrdU Cell Proliferation ELISA Kit (Roche, Shanghai, China) as previously described [47].

### Glucose consumption

Cells were seeded in culture dishes and the media were changed after 6 h. The cells were further incubated for 24 h, and the culture media were collected and used for glucose measurement using a Glucose Assay kit (Sigma-Aldrich, Shanghai, China). The assay was performed according to the manufacturer's instructions. The glucose consumption and lactate production were normalized by cell counts (per 10^6^). First, 1 mL of culture medium of each sample was added into a test tube and a standard sample in another tube was prepared by adding 950 μL of water and 50 μL of the Glucose Standard solution into the tube. The reactions were started at zero time by adding 2 mL of the Assay Reagent to each tube at an interval of 30–60 seconds. The tubes were incubated at 37°C for 30 min. The reactions were stopped by adding 2 mL of 12 N H_2_SO_4_ into each tube at 30–60 seconds intervals. The absorbance of each tube against the reagent blank at 540 nm was measured using a microplate spectrophotometer. The glucose concentration was calculated as: mg glucose = (ΔA_540_ of Test) (mg glucose in Standard)/ΔA_540_ of Standard. The glucose consumption was: (original glucose concentration in the medium) – (glucose concentration at test).

### Growth curve assay

Cell proliferation was measured using methylthiazolyldiphenyl-tetrazolium bromide (MTT) assay as previously described [46]. Alternatively, cells were counted by a Vi-Cell XR cell viability analyzer (Beckman Coulter). Inhibition assays were performed by adding 1 nmol/L mk2206 dihydrochloride (Selleck Chemicals, Shanghai, China) or 10 or 25 μM LY294002 (Selleck Chemicals, Shanghai, China) to the culture medium.

### Luciferase reporter assay

The promoter regions of *PIK3CA* and *PDK1* was amplified using normal human genomic DNA, and inserted into pGL3 luciferase reporter vector (Promega, Shanghai, China) [22]. Three promoter regions of *PIK3CA* were cloned, including −1889 to +1 (transcriptional start), −1276 to +1 and −516 to +1. The promoter region from −1325 to +1 (transcriptional start) of *PDK1* was cloned for analysis. The primers were deposited in Supplementary Table 1. The procedure has been described previously [22].

### ChIP assay

ChIP assay was performed using an antibody against BRG1 or IgG as a negative control with an EZ-ChIP kit (Upstate Biotechnology) according to the manufacturer's instruction and previous reports [22, 48]. The primers used to amplify the promoter fragments were listed in Supplementary Table 1.

### Drug sensitivity assay

Gastric cancer cells (1–2 × 10^4^ per well) were seeded on a 96-well plate and treated with different concentrations of drug chemicals for 72 hrs at 37°C. The concentrations of LY294002 and AT7867 (Selleck Chemicals, Shanghai, China) were 0, 0.01, 0.1, 1, 10 and 100 μM. Twenty μL of MTT solution (5 mg/mL) (Sigma-Aldrich, Shanghai, China) was added to each well and the cells were incubated for 4 hrs at 37°C. Finally, 150 μL DMSO was added and allowed to dissolve for 10 min. The absorbance was monitored at 490 nm using a microplate spectrophotometer.

### Xenograft mouse transplantation model

The BK nude mice (male) were 4–5 weeks old at the beginning of the study. These mice were purchased from Shanghai Laboratory Animal Center, Shanghai, China, and allowed to acclimate for 5 days in the animal facility before further intervention. Cells (5 × 10^6^) mixed with equal volume of Matrigel (BD Biosciences, Shanghai, China) were injected subcutaneously into both flanks of each mouse. When the tumor volume reached 100 mm^3^, the mice were injected intraperitoneally of LY294002 (Selleck Chemicals, Shanghai, China) every day with a dosage of 30 mg/kg (body weight). The control group was injected with 1 × PBS (pH 7.4). Tumor diameters were measured using a caliper every two days and the tumor volume was calculated using the formula: V (mm^3^) = (width)^2^ × length/2. The mice were executed 30 days after cell inoculation. The tumors were isolated from the bodies and weighed, followed by fixation with 4% neutral paraformaldehyde. All experimental procedures were conducted according to the guidelines of the Animal Experimental Ethics Committee of Shanghai Medical College of Fudan University.

### Statistics

Differential significance was calculated using unpaired Student's *t* test. Drug sensitivity statistics was performed using GraphPad Prism 6 and the concentration was Log transformed and the divergence of LogIC50 was evaluated with an F-test. A *p* value < 0.05 was considered as statistically significant. All statistical tests were two-sided.

## SUPPLEMENTARY FIGURES AND TABLES





## Abbreviations

ARID: AT-rich interactive domain
ARID1A-fl: full-length ARID1A
ARID1A: AT-rich interactive domain-containing protein 1A
BAD: Bcl-2-associated death promoter
BAF250a: BRG1-associated factor 250 a
BCL-2: B-cell lymphoma 2
BRG1: BRM/SWI2-related gene 1
EMT: epithelial-mesenchymal transition
GSK3α/β: glycogen synthase kinase 3α/β
GC: Gastric cancer
GR: glucocorticoid receptor
HIC1: Hypermethylated in cancer 1
mTOR: mammalian target of rapamycin
OCCC: ovarian clear-cell carcinomas
SWI/SNF: SWItch/Sucrose Non-Fermentable.

## References

[1] Wang K, Kan J, Yuen ST, Shi ST, Chu KM, Law S, Chan TL, Kan Z, Chan AS, Tsui WY, Lee SP, Ho SL, Chan AK (2011). Exome sequencing identifies frequent mutation of ARID1A in molecular subtypes of gastric cancer. Nat Genet.

[2] Kim TM, Jung SH, Kim MS, Baek IP, Park SW, Lee SH, Lee HH, Kim SS, Chung YJ, Lee SH (2014). The mutational burdens and evolutionary ages of early gastric cancers are comparable to those of advanced gastric cancers. J Pathol.

[3] Chen K, Yang D, Li X, Sun B, Song F, Cao W, Brat DJ, Gao Z, Li H, Liang H, Zhao Y, Zheng H, Li M (2015). Mutational landscape of gastric adenocarcinoma in Chinese: Implications for prognosis and therapy. Proc Natl Acad Sci U S A.

[4] Wang K, Yuen ST, Xu J, Lee SP, Yan HH, Shi ST, Siu HC, Deng S, Chu KM, Law S, Chan KH, Chan AS, Tsui WY (2014). Whole-genome sequencing and comprehensive molecular profiling identify new driver mutations in gastric cancer. Nat Genet.

[5] Zang ZJ, Cutcutache I, Poon SL, Zhang SL, McPherson JR, Tao J, Rajasegaran V, Heng HL, Deng N, Gan A, Lim KH, Ong CK, Huang D (2012). Exome sequencing of gastric adenocarcinoma identifies recurrent somatic mutations in cell adhesion and chromatin remodeling genes. Nat Genet.

[6] Jones S, Wang TL, Shih Ie M, Mao TL, Nakayama K, Roden R, Glas R, Slamon D, Diaz LA, Vogelstein B, Kinzler KW, Velculescu VE, Papadopoulos N (2010). Frequent mutations of chromatin remodeling gene ARID1A in ovarian clear cell carcinoma. Science.

[7] Kandoth C, Schultz N, Cherniack AD, Akbani R, Liu Y, Shen H, Robertson AG, Pashtan I, Shen R, Benz CC, Yau C, Laird PW, Cancer Genome Atlas Research N (2013). Integrated genomic characterization of endometrial carcinoma. Nature.

[8] Liang H, Cheung LW, Li J, Ju Z, Yu S, Stemke-Hale K, Dogruluk T, Lu Y, Liu X, Gu C, Guo W, Scherer SE, Carter H (2012). Whole-exome sequencing combined with functional genomics reveals novel candidate driver cancer genes in endometrial cancer. Genome Res.

[9] Giulino-Roth L, Wang K, MacDonald TY, Mathew S, Tam Y, Cronin MT, Palmer G, Lucena-Silva N, Pedrosa F, Pedrosa M, Teruya-Feldstein J, Bhagat G, Alobeid B (2012). Targeted genomic sequencing of pediatric Burkitt lymphoma identifies recurrent alterations in antiapoptotic and chromatin-remodeling genes. Blood.

[10] Jiang L, Gu ZH, Yan ZX, Zhao X, Xie YY, Zhang ZG, Pan CM, Hu Y, Cai CP, Dong Y, Huang JY, Wang L, Shen Y (2015). Exome sequencing identifies somatic mutations of DDX3X in natural killer/T-cell lymphoma. Nat Genet.

[11] Zhang J, Grubor V, Love CL, Banerjee A, Richards KL, Mieczkowski PA, Dunphy C, Choi W, Au WY, Srivastava G, Lugar PL, Rizzieri DA, Lagoo AS (2013). Genetic heterogeneity of diffuse large B-cell lymphoma. Proc Natl Acad Sci U S A.

[12] Guichard C, Amaddeo G, Imbeaud S, Ladeiro Y, Pelletier L, Maad IB, Calderaro J, Bioulac-Sage P, Letexier M, Degos F, Clement B, Balabaud C, Chevet E (2012). Integrated analysis of somatic mutations and focal copy-number changes identifies key genes and pathways in hepatocellular carcinoma. Nat Genet.

[13] Huang J, Deng Q, Wang Q, Li KY, Dai JH, Li N, Zhu ZD, Zhou B, Liu XY, Liu RF, Fei QL, Chen H, Cai B (2012). Exome sequencing of hepatitis B virus-associated hepatocellular carcinoma. Nat Genet.

[14] Fujimoto A, Totoki Y, Abe T, Boroevich KA, Hosoda F, Nguyen HH, Aoki M, Hosono N, Kubo M, Miya F, Arai Y, Takahashi H, Shirakihara T (2012). Whole-genome sequencing of liver cancers identifies etiological influences on mutation patterns and recurrent mutations in chromatin regulators. Nat Genet.

[15] Imielinski M, Berger AH, Hammerman PS, Hernandez B, Pugh TJ, Hodis E, Cho J, Suh J, Capelletti M, Sivachenko A, Sougnez C, Auclair D, Lawrence MS (2012). Mapping the hallmarks of lung adenocarcinoma with massively parallel sequencing. Cell.

[16] Seo JS, Ju YS, Lee WC, Shin JY, Lee JK, Bleazard T, Lee J, Jung YJ, Kim JO, Shin JY, Yu SB, Kim J, Lee ER (2012). The transcriptional landscape and mutational profile of lung adenocarcinoma. Genome Res.

[17] Cancer Genome Atlas Research N (2014). Comprehensive molecular profiling of lung adenocarcinoma. Nature.

[18] Roberts CW, Orkin SH (2004). The SWI/SNF complex--chromatin and cancer. Nat Rev Cancer.

[19] Wu JN, Roberts CW (2013). ARID1A mutations in cancer: another epigenetic tumor suppressor?. Cancer Discov.

[20] Guan B, Wang TL, Shih Ie M (2011). ARID1A, a factor that promotes formation of SWI/SNF-mediated chromatin remodeling, is a tumor suppressor in gynecologic cancers. Cancer Res.

[21] Wang DD, Chen YB, Pan K, Wang W, Chen SP, Chen JG, Zhao JJ, Lv L, Pan QZ, Li YQ, Wang QJ, Huang LX, Ke ML (2012). Decreased expression of the ARID1A gene is associated with poor prognosis in primary gastric cancer. PLoS One.

[22] Yan HB, Wang XF, Zhang Q, Tang ZQ, Jiang YH, Fan HZ, Sun YH, Yang PY, Liu F (2014). Reduced expression of the chromatin remodeling gene ARID1A enhances gastric cancer cell migration and invasion via downregulation of E-cadherin transcription. Carcinogenesis.

[23] Kim MS, Je EM, Yoo NJ, Lee SH (2012). Loss of ARID1A expression is uncommon in gastric, colorectal, and prostate cancers. APMIS.

[24] Wiegand KC, Sy K, Kalloger SE, Li-Chang H, Woods R, Kumar A, Streutker CJ, Hafezi-Bakhtiari S, Zhou C, Lim HJ, Huntsman DG, Clarke B, Schaeffer DF (2014). ARID1A/BAF250a as a prognostic marker for gastric carcinoma: a study of 2 cohorts. Hum Pathol.

[25] Mamo A, Cavallone L, Tuzmen S, Chabot C, Ferrario C, Hassan S, Edgren H, Kallioniemi O, Aleynikova O, Przybytkowski E, Malcolm K, Mousses S, Tonin PN (2012). An integrated genomic approach identifies ARID1A as a candidate tumor-suppressor gene in breast cancer. Oncogene.

[26] Van Rechem C, Boulay G, Leprince D (2009). HIC1 interacts with a specific subunit of SWI/SNF complexes, ARID1A/BAF250A. Biochem Biophys Res Commun.

[27] Nagl NG, Zweitzig DR, Thimmapaya B, Beck GR, Moran E (2006). The c-myc gene is a direct target of mammalian SWI/SNF-related complexes during differentiation-associated cell cycle arrest. Cancer Res.

[28] Nagl NG, Wang X, Patsialou A, Van Scoy M, Moran E (2007). Distinct mammalian SWI/SNF chromatin remodeling complexes with opposing roles in cell-cycle control. EMBO J.

[29] Chandler RL, Damrauer JS, Raab JR, Schisler JC, Wilkerson MD, Didion JP, Starmer J, Serber D, Yee D, Xiong J, Darr DB, de Villena FP, Kim WY (2015). Coexistent ARID1A-PIK3CA mutations promote ovarian clear-cell tumorigenesis through pro-tumorigenic inflammatory cytokine signalling. Nat Commun.

[30] Anglesio MS, Bashashati A, Wang YK, Senz J, Ha G, Yang W, Aniba MR, Prentice LM, Farahani H, Li Chang H, Karnezis AN, Marra MA, Yong PJ (2015). Multifocal endometriotic lesions associated with cancer are clonal and carry a high mutation burden. J Pathol.

[31] Huang HN, Lin MC, Huang WC, Chiang YC, Kuo KT (2014). Loss of ARID1A expression and its relationship with PI3K-Akt pathway alterations and ZNF217 amplification in ovarian clear cell carcinoma. Mod Pathol.

[32] Yamamoto S, Tsuda H, Takano M, Tamai S, Matsubara O (2012). Loss of ARID1A protein expression occurs as an early event in ovarian clear-cell carcinoma development and frequently coexists with PIK3CA mutations. Mod Pathol.

[33] Zeng Y, Liu Z, Yang J, Liu Y, Huo L, Li Z, Lan S, Wu J, Chen X, Yang K, Li C, Li M, Liu J (2013). ARID1A is a tumour suppressor and inhibits glioma cell proliferation via the PI3K pathwa. Head Neck Oncol.

[34] Wiegand KC, Hennessy BT, Leung S, Wang Y, Ju Z, McGahren M, Kalloger SE, Finlayson S, Stemke-Hale K, Lu Y, Zhang F, Anglesio MS, Gilks B (2014). A functional proteogenomic analysis of endometrioid and clear cell carcinomas using reverse phase protein array and mutation analysis: protein expression is histotype-specific and loss of ARID1A/BAF250a is associated with AKT phosphorylation. BMC Cancer.

[35] Xie C, Fu L, Han Y, Li Q, Wang E (2014). Decreased ARID1A expression facilitates cell proliferation and inhibits 5-fluorouracil-induced apoptosis in colorectal carcinoma. Tumour Biol.

[36] Wilson BG, Roberts CW (2011). SWI/SNF nucleosome remodellers and cancer. Nat Rev Cancer.

[37] Nie Z, Xue Y, Yang D, Zhou S, Deroo BJ, Archer TK, Wang W (2000). A specificity and targeting subunit of a human SWI/SNF family-related chromatin-remodeling complex. Mol Cell Biol.

[38] Wilsker D, Patsialou A, Zumbrun SD, Kim S, Chen Y, Dallas PB, Moran E (2004). The DNA-binding properties of the ARID-containing subunits of yeast and mammalian SWI/SNF complexes. Nucleic Acids Res.

[39] Samartzis EP, Gutsche K, Dedes KJ, Fink D, Stucki M, Imesch P (2014). Loss of ARID1A expression sensitizes cancer cells to PI3K- and AKT-inhibition. Oncotarget.

[40] Kato S, Schwaederle M, Daniels GA, Piccioni D, Kesari S, Bazhenova L, Shimabukuro K, Parker BA, Fanta P, Kurzrock R (2015). Cyclin-dependent kinase pathway aberrations in diverse malignancies: clinical and molecular characteristics. Cell Cycle.

[41] Berthelet J, Dubrez L (2013). Regulation of Apoptosis by Inhibitors of Apoptosis (IAPs). Cells.

[42] Zhou BP, Liao Y, Xia W, Zou Y, Spohn B, Hung MC (2001). HER-2/neu induces p53 ubiquitination via Akt-mediated MDM2 phosphorylation. Nat Cell Biol.

[43] Zhai Y, Kuick R, Tipton C, Wu R, Sessine M, Wang Z, Baker SJ, Fearon ER, Cho KR (2016). Arid1a inactivation in an Apc and Pten-defective mouse ovarian cancer model enhances epithelial differentiation and prolongs survival. J Pathol.

[44] Zhang W, Zeng X, Briggs KJ, Beaty R, Simons B, Chiu Yen RW, Tyler MA, Tsai HC, Ye Y, Gesell GS, Herman JG, Baylin SB, Watkins DN (2010). A potential tumor suppressor role for Hic1 in breast cancer through transcriptional repression of ephrin-A1. Oncogene.

[45] Sastry L, Johnson T, Hobson MJ, Smucker B, Cornetta K (2002). Titering lentiviral vectors: comparison of DNA, RNA and marker expression methods. Gene Ther.

[46] Chen SX, Xu XE, Wang XQ, Cui SJ, Xu LL, Jiang YH, Zhang Y, Yan HB, Zhang Q, Qiao J, Yang PY, Liu F (2014). Identification of colonic fibroblast secretomes reveals secretory factors regulating colon cancer cell proliferation. J Proteomics.

[47] Qiao J, Cui SJ, Xu LL, Chen SJ, Yao J, Jiang YH, Peng G, Fang CY, Yang PY, Liu F (2015). Filamin C, a dysregulated protein in cancer revealed by label-free quantitative proteomic analyses of human gastric cancer cells. Oncotarget.

[48] Cascio S, Zhang L, Finn OJ (2011). MUC1 protein expression in tumor cells regulates transcription of proinflammatory cytokines by forming a complex with nuclear factor-kappaB p65 and binding to cytokine promoters: importance of extracellular domain. J Biol Chem.

