# Supplementary material for: Chromatin remodeling gene AT-rich interactive domain-containing protein 1A suppresses gastric cancer cell proliferation by targeting PIK3CA and PDK1
Source: Oncotarget. 2016 Jun 15;7(29):46127–41. doi: 10.18632/oncotarget.10060 (PMC5216786; doi:10.18632/oncotarget.10060)
Supplement: Supplementary file 1 [file oncotarget-07-46127-s001.pdf]

Chromatin remodeling gene AT-rich interactive domain-containing protein 1A suppresses gastric cancer cell proliferation by targeting *PIK3CA* and *PDK1*

SUPPLEMENTARY FIGURES AND TABLES

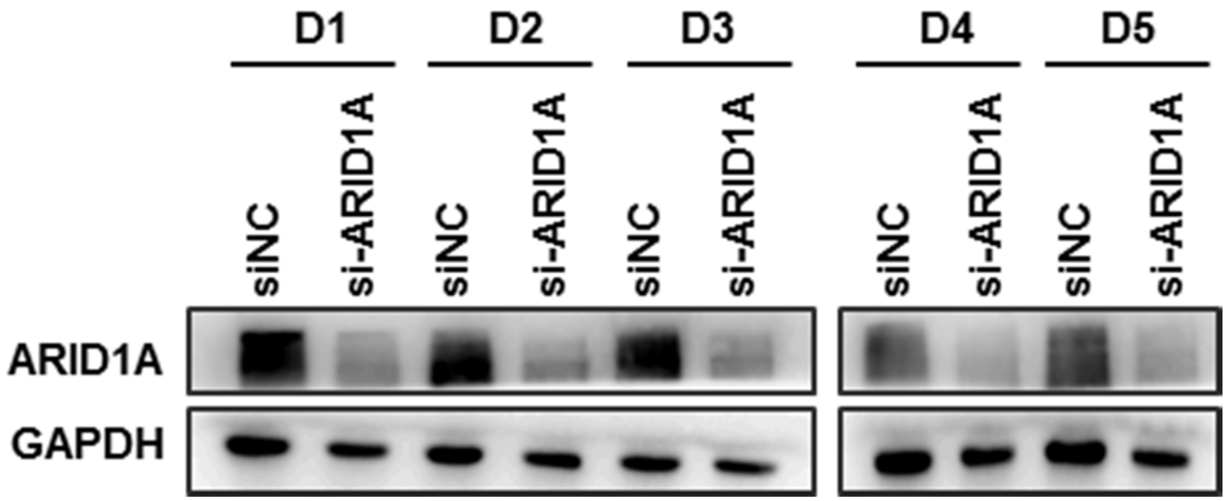

Supplementary Figure S1: Western blot analysis of ARID1A expression in SGC-7901 cells after silencing with a siRNA at different time point. SGC-7901 cells were transfected with a siRNA targeting *ARID1A* after plating for 24 hrs. After a further culture of 24 hrs, the cells were seeded onto a 96-well plate for growth assay. siNC, negative control of transfection by a scramble siRNA. D1, 24 hrs after transfection.

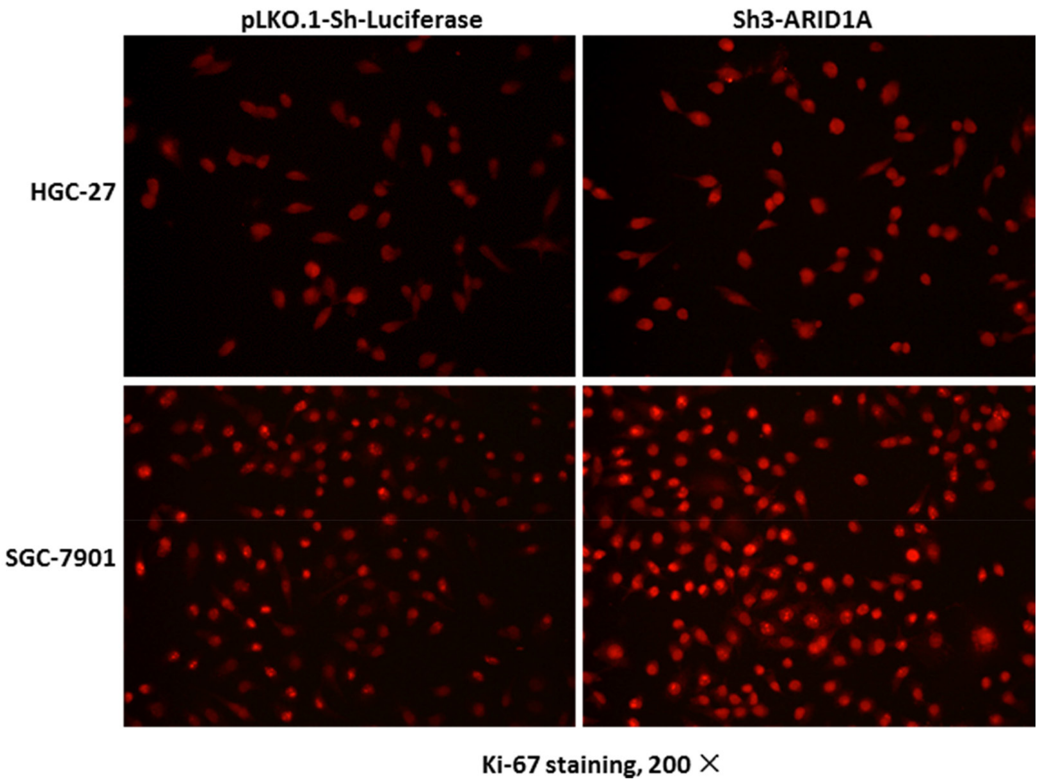

Supplementary Figure S2: Ki-67 staining of gastric cancer cells after *ARID1A* was silenced with a shRNA. Sh-Luciferase was served as a negative control. The exposure was set at the same condition throughout all images.

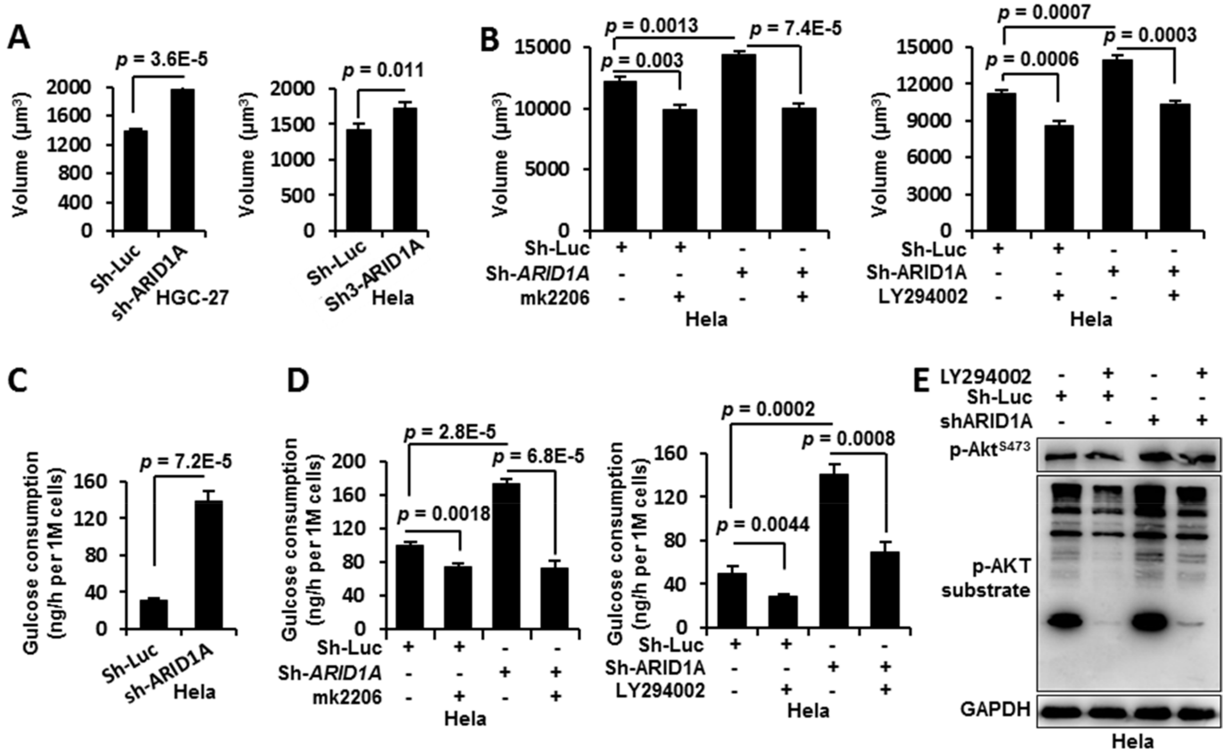

Supplementary Figure S3: *ARID1A* silencing promotes GC cell growth by targeting PI3K/AKT pathway. A. *ARID1A* was silenced in HGC-27 and Hela cells and cell volume was measured. **B.** Hela cell volume was increased by *ARID1A* silencing but was reduced by mk2206 and LY294002 treatment. **C.** Glucose consumption was increased in Hela cells by *ARID1A* knockdown. **D.** Glucose uptake was increased by *ARID1A* silencing but was inhibited by mk2206 and LY294002 treatment. **E.** The activity of the substrate of p-AKT was analyzed in Hela cells with *ARID1A*-deficiency.

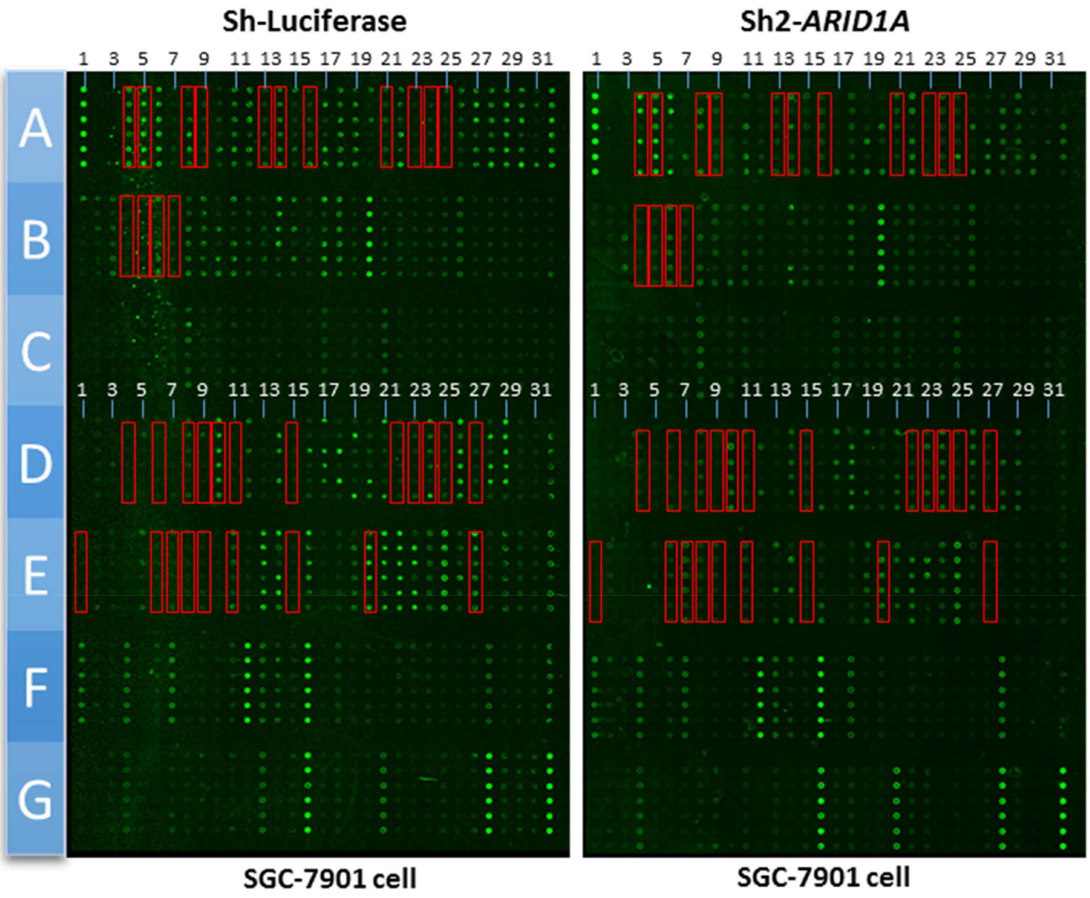

Supplementary Figure S4: AKT/PKB phospho antibody array analysis of gastric cancer cells with *ARID1A* silencing. Sh-Luciferase was served as a negative control. The immno reaction and scanning conditions for the two arrays were set at the same condition. The spots highlighted with red blocks were phosphorylated proteins with prominent changes.

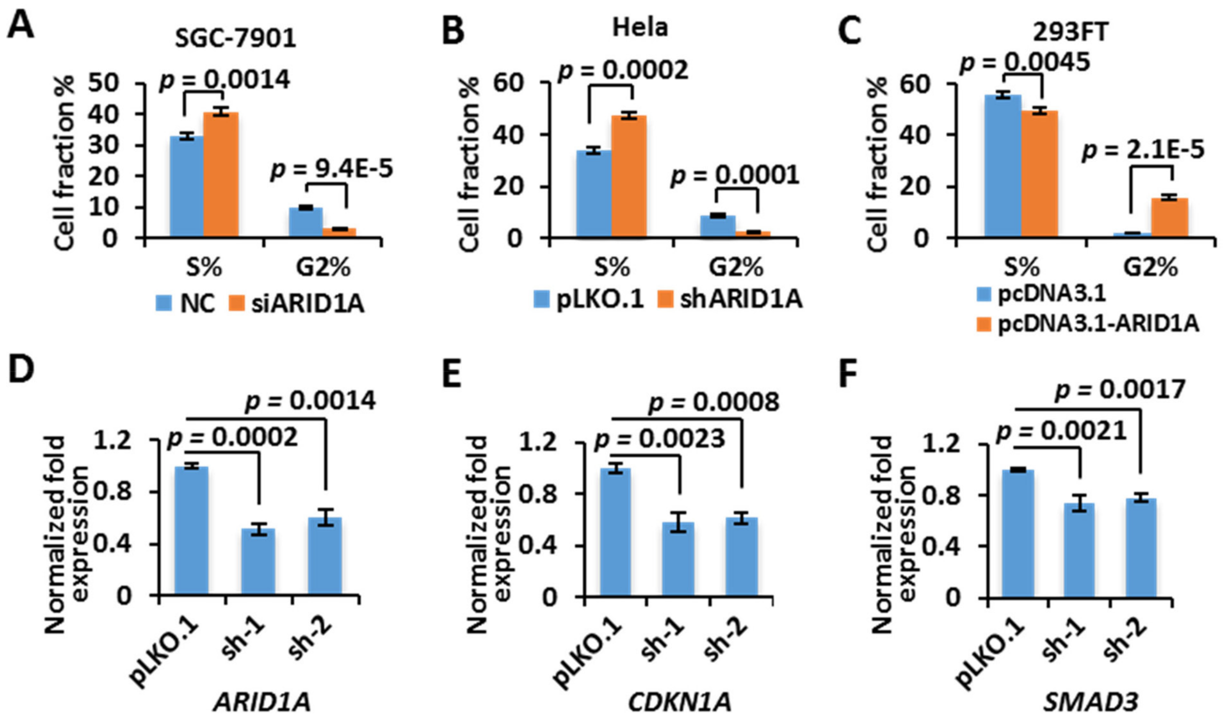

Supplementary Figure S5: The modulation role of gastric cancer cell cycle by *ARID1A*. A. *ARID1A* was silenced in SGC-7901 by a siRNA and the cell cycle changes were measured using flow cytometry. NC, negative control using a scrambled siRNA. **B.** *ARID1A* was depleted in Hela cell with a shRNA and the cell cycle changes were analyzed. pLKO.1 indicates a control silencing against luciferase. **C.** *ARID1A* was ectopically expressed in 293FT cell and the cell cycle changes were analyzed. *p* values were calculated using two-sided Student's *t* test based on triplicate experiments and a p value < 0.05 was considered as statistically significant. **D.** qPCR analysis of *ARID1A* in HGC-27 cells stably transfected with shRNAs against *ARID1A* (sh1 and sh2). pLKO.1 indicates a control silencing against luciferase. **E.** qPCR analysis of the expression of *cyclin-dependent kinase inhibitor 1A* (*CDKN1A*), which encodes a tumor suppressor, p21. **F.** qPCR analysis of *SMAD family member 3* (*SMAD3*) after *ARID1A* was silenced in HGC-27 cells. *p* values were calculated using two-sided Student's *t* test based on triplicate experiments and a p value < 0.05 was considered as statistically significant.

Supplementary Table S1: Primers, siRNAs and shRNAs

See Supplementary File 1

Supplementary Table S2: Antibodies used in current study

Cat No.	Company	Antibody	Clone	Animal host
9234	CST	PHOSPHO-p70S6K(Thr389)	108D2	Rabbit
2708	CST	p70 S6 Kinase	49D7	Rabbit
2971	CST	Phospho-mTOR (Ser2448)	-	Rabbit
2972	CST	mTOR	-	Rabbit
8566	CST	Phospho-GSK-3α/β (Ser21/9)	D17D2	Rabbit
5676	CST	GSK-3α/β	D75D3	Rabbit
4060	CST	Phospho-Akt (Ser473)	D9E	Rabbit
4056	CST	Phospho-Akt (Thr308)	244F9	Rabbit
9611	CST	Phospho-(Ser/Thr) Akt Substrate Antibody	-	Rabbit
4691	CST	Akt (pan)	C67E7	Rabbit
2947	CST	p21 Waf1/Cip1	12D1	Rabbit
3061	CST	Phospho-PDK1 (Ser241)	-	Rabbit
3062	CST	PDK1	-	Rabbit
18262-1-AP	PTG lab	PDK1	-	Rabbit
20583-1-AP	PTG lab	PI3K	-	Rabbit
21829-1-AP	PTG lab	GLUT1	-	Rabbit
SC-7938	Santa Cruz	GLUT4	H-61	Rabbit
sc-10768	Santa Cruz	BRG1	H-88	Rabbit
HPA005456	Sigma	ARID1A	-	Rabbit
sc-23900	Santa Cruz	Ki-67	-	Mouse
06912c	CWBIO	GAPDH	-	Mouse
66008-1-Ig	PTG lab	Flag	-	Mouse
sc-2005	Santa Cruz	goat anti-mouse IgG-HRP	-	Goat
sc-2004	Santa Cruz	goat anti-rabbit IgG-HRP	-	Goat

Supplementary Table S3: Phospho-protein antibody array analysis of SGC-7901 cells with *ARID1A* silencing

Block[1]	Column[1]	Name	Sh-Luciferase[2]	Sh2-*ARID1A*[2]	Fold of change	*p* value[3]
5	8	14-3-3 theta/tau (Ser232)	76.33	277.24	3.63	3.40E-07
5	9	14-3-3 zeta/delta (Thr232)	84.83	272.54	3.21	3.15E-05
1	8	AKT (Ser473)	261.67	882.62	3.37	3.50E-04
1	9	AKT (Thr308)	83.17	433.24	5.21	2.13E-08
4	9	AKT1 (Ser124)	34.40	91.63	2.66	3.07E-04
4	8	AKT1 (Thr450)	125.00	335.98	2.69	3.23E-02
4	15	AKT1S1 (Thr246)	68.33	285.70	4.18	9.06E-10
5	15	BAD (Ser134)	63.67	156.95	2.47	1.26E-07
1	16	BAD (Ser136)	110.60	430.90	3.90	5.23E-06
1	14	BCL-2 (Ser70)	247.40	571.39	2.31	1.90E-02
1	13	BCL-2 (Thr56)	121.00	343.42	2.84	5.13E-04
5	11	BCL-2 (Thr69)	202.67	367.30	1.81	9.04E-03
4	10	Cyclin D1 (Thr286)	413.00	874.01	2.12	4.66E-05
4	11	FAK (Tyr576)	47.17	234.01	4.96	1.66E-07
1	4	GSK3 beta (Ser9)	535.60	1426.14	2.66	6.53E-05
4	4	GSK3α-β (Tyr216/279)	35.50	245.52	6.92	3.10E-11
4	22	MDM2 (Ser166)	84.83	338.33	3.99	3.46E-06
2	7	mTOR (Ser2448)	92.83	278.65	3.00	7.50E-09
5	6	mTOR (Ser2481)	57.67	277.63	4.81	9.03E-04
5	19	mTOR (Thr2446)	199.40	535.68	2.69	4.26E-03
2	4	p21Cip1 (Thr145)	70.17	184.43	2.63	2.97E-06
2	5	p27Kip1 (Ser10)	68.50	336.92	4.92	7.28E-08
2	6	p27Kip1 (Thr187)	297.50	716.59	2.41	3.37E-05
1	21	p53 (Ser15)	123.33	395.10	3.20	3.55E-03
4	23	p53 (Ser20)	100.67	450.32	4.47	6.53E-04
1	23	p53 (Ser33)	55.50	113.72	2.05	8.73E-07
1	24	p53 (Ser37)	105.40	419.38	3.98	6.83E-05
4	24	p53 (Ser392)	190.17	659.42	3.47	4.90E-05
1	25	p53 (Ser46)	70.00	304.96	4.36	2.18E-06
5	7	p53 (Thr81)	109.00	597.71	5.48	2.51E-07
4	25	p70S6K (Thr229)	93.60	204.41	2.18	5.22E-05
4	27	p70S6K (Thr389)	99.83	271.60	2.72	2.92E-05
4	6	p70S6K (Thr421)	61.20	250.61	4.09	7.83E-03
1	5	PDK1 (Ser241)	615.50	1427.31	2.32	6.26E-05
5	1	Tuberin/TSC2 (Thr1462)	36.67	70.09	1.91	1.29E-02
5	27	XIAP (Ser87)	347.00	124.05	0.36	1.95E-04

The experiment was performed by Wayen Biotechnologies (Shanghai), Inc., using the Full Moon AKT/PKB phospho antibody array (Full Moon BioSystems, Inc.).

[1]The block and column define the position of spots of proteins analyzed. These positions were highlighted in Supplementary Figure 4.

[2]The values were normalization with actin.

[3]The p value was calculated using two-sided Student's *t* test and $p < 0.05$ was considered as statistically significant.
