# Supplementary material for: Chromatin remodeling gene AT-rich interactive domain-containing protein 1A suppresses gastric cancer cell proliferation by targeting PIK3CA and PDK1
Source: Oncotarget. 2016 Jun 15;7(29):46127–41. doi: 10.18632/oncotarget.10060 (PMC5216786; doi:10.18632/oncotarget.10060)
Supplement: Supplementary file 2 [file oncotarget-07-46127-s002.docx]

Supplementary Table S1**.** Primers, siRNAs and shRNAs.

^1^siRNAs of ARID1A were synthesized according to Wang, *et al*. (PloS one 2012, 7: e40364.)

^2^The short hairpin RNA (shRNA) lentiviral plasmid (pLKO.1) was obtained from the RNA interference (RNAi) consortium.

| **Category** | **Name** | **Sequences** | **Vector** |
| --- | --- | --- | --- |
| **Cloning primer** | | | |
|  | **ARID1A-fl (full length)** |  |  |
|  | ARID1A-Nhe1-F | ATGGGTTCTAGAGAGCTAGCGCCACCATGG | pcDNA3.1/myc-His(-) A |
|  | ARID1A-Xho1-R | GCCCTCGAGGCTCATGACTGGCCAATCAAAAACAGT | pcDNA3.1/myc-His(-) A |
|  | ARID1A-Mfe1-F | GTACAATTG ATGGCCGCGCAGGTCGC | pPB-CAG |
|  | ARID1A-Xho1-R | GACTCGAGTCATGACTGGCCAATCAA | pPB-CAG |
|  | N-ARID1A--Bcl1-F | ATGGGTTCTAGAGATGATCAGCCACCATGG | pBABE |
|  | C-ARID1A-Xho1-R | GCCCTCGAGGCTCATGACTGGCCAATCAAAAACAGT | pBABE |
|  | ARID1A-Bcl1-F | AGATGATCATATGGCCGCGCAGGTCGC | pCMV |
|  | ARID1A-Xho1-R | GCCCTCGAGGCTCATGACTGGCCAATCAAAAACAGT | pCMV |
|  | **ARID1A-c1** |  |  |
|  | C-terminal-PPB-mfe1-ARID1A-F | GCACAATTGAATTCCATGACTCCAAAC | pPB-CAG |
|  | C-terminal-PPB-Xho1-ARID1A-R | GACTCGAGTCATGACTGGCCAATCAA | pPB-CAG |
|  | C-terminal-ARID1A--EcoRI-ATG-F | GA GAATTC GCCACC ATG GC GTCCATGACTCCAAACCCTG | pBABE |
|  | C-terminal-ARID1A-Sal1-R | GCC GTCGAC TCATGACTGGCCAATCAAAAACAGT | pBABE |
|  | **ARID1A-c2** |  |  |
|  | ARID1A-EcoR-F | GAGAATTCGATGCTGCACACAGATCAGAG | pCMV-TAG2A |
|  | ARID1A-Sal1-R | GCCGTCGACTCATGACTGGCCAATCAAAA | pCMV-TAG2A |
|  | **ARID1A-c3** |  |  |
|  | ARID1A-EcoR-F | GAGAATTCGCTACTGGATCCTGGGAGGTT | pCMV-TAG2A |
|  | ARID1A-Sal1-R | GCCGTCGACTCATGACTGGCCAATCAAAA | pCMV-TAG2A |
|  | **ARID1A-c4** |  |  |
|  | ARID1A-EcoR-F | GAGAATTCGACAACAGCAGAGGGTACAC | pCMV-TAG2A |
|  | ARID1A-Sal1-R | GCCGTCGACTCATGACTGGCCAATCAAAA | pCMV-TAG2A |
| **qPCR primer** | | | |
|  | ARID1A-F | CAGTACCTGCCTCGCACATA | - |
|  | ARID1A-R | GCCAGGAGACCAGACTTGAG | - |
|  | PTEN-F | TGGAAAGGGACGAACTGGTG | - |
|  | PTEN-R | CATAGCGCCTCTGACTGGGA | - |
|  | PDK1-F | AAGATGAGTGACCGAGGAGGT | - |
|  | PDK1-R | CCATAACCAAAACCAGCCAGAG | - |
|  | PIK3CA-F | CCCCTCCATCAACTTCTTCA | - |
|  | PIK3CA-R | CGGTTGCCTACTGGTTCAAT | - |
|  | CDKN1A-F | GTGTGAGCAGCTGCCGAAGTCA | - |
|  | CDKN1A-R | TGACATGGCGCCTCCTCTGAGT | - |
|  | SMAD3-F | TCAACACCAAGTGCATCACC | - |
|  | SMAD3-R | CGGCAGTAGATGACATGAGG | - |
|  | GAPDH-F | CGAGATCCCTCCAAAATCAA | - |
|  | GAPDH-R | TTCACACCCATGACGAACAT | - |
| **Promoter cloning primer (Report assay)** | | | |
|  | PIK3CA promoter-(full length)-F | GAGGTACCCGGCTGAAGTCTGTAATCCCAACA | pGL3 |
|  | PIK3CA promoter-(full length)-R | AGTCTCGAGTTATTGGTCTCTTTTTCCGCTCACATC | pGL3 |
|  | PIK3CA promoter 1-F | GAGGTACCGAGTAACAGTGTTCTAAAACTGT | pGL3 |
|  | PIK3CA promoter 1-R | AGTCTCGAGTTATTGGTCTCTTTTTCCGCTCACATC | pGL3 |
|  | PIK3CA promoter 2-F | GAGGTACCTCGGCAGAAGAACGCACAGCAA | pGL3 |
|  | PIK3CA promoter 2-R | AGTCTCGAGTTATTGGTCTCTTTTTCCGCTCACATC | pGL3 |
|  | PDK1 promoter-(full length)-F | GAGGTACCGGAAAAACCTGCCTCCCATTC | pGL3 |
|  | PDK1 promoter-(full length)-R | AGTCTCGAGAAGCTTCCCGCTAGAGAAGCC | pGL3 |
| **siRNA** | | | |
|  | siRNA-ARID1A-sense^1^ | GCCCUAACAUGGCCAAUAUTT | - |
|  | siRNA-ARID1A-antisense^1^ | AUAUUGGCCAUGUUAGGGCTT | - |
|  | Negative control-sense | UUCUCCGAACGUGUCACGUTT | - |
|  | Negative control-antisense | ACGUGACACGUUCGGAGAATT | - |
| **shRNA^2^** | | | |
|  | ARID1A sh1 | GCCTGATCTATCTGGTTCAAT | TRCN0000059089 |
|  | ARID1A sh2 | CCTCTCTTATACACAGCAGAT | TRCN0000059090 |
|  | ARID1A sh3 | CCTCTCTTATACACAGCAGAT | TRCN0000059091 |
